# Perforated Amyand hernia with an adenocarcinoma tumour presenting as a groin abscess

**DOI:** 10.1093/bjrcr/uaae008

**Published:** 2024-03-06

**Authors:** Emmanuel Gbegli, Ahmad Miremadi, Eva Mendes Serrao, Timothy J Sadler

**Affiliations:** Department of Radiology, Addenbrooke’s Hospital, Cambridge University Hospitals NHS Foundation Trust, Cambridge CB2 0QQ, United Kingdom; Department of Histopathology, Addenbrooke’s Hospital, Cambridge University Hospitals NHS Foundation Trust, Cambridge CB2 0QQ, United Kingdom; Department of Radiology, Addenbrooke’s Hospital, Cambridge University Hospitals NHS Foundation Trust, Cambridge CB2 0QQ, United Kingdom; Department of Radiology, University of Cambridge, Cambridge CB2 0QQ, United Kingdom; Department of Radiology, Addenbrooke’s Hospital, Cambridge University Hospitals NHS Foundation Trust, Cambridge CB2 0QQ, United Kingdom

**Keywords:** Amyand hernia, hernia, appendicitis, perforation, adenocarcinoma, abscess, ultrasound, CT

## Abstract

An Amyand hernia is an incarcerated inguinal hernia containing the appendix with or without appendicitis. This is a rare form of inguinal hernia, making up approximately 0.4%-1% of all cases. As with any hernia, this may become strangulated at any time, leading to the loss of blood supply and further development of gangrene and complications. Clinically, this can present in a manner indistinguishable from other types of inguinal hernias. In addition, the appendix can be affected by its own set of pathological processes, such as infection, inflammation, and malignancy. Not uncommonly both hernial and appendiceal complications coexist. The clinical diagnosis of an Amyand hernia remains challenging due to its low incidence and indistinct clinical presentation. At present, surgery is usually diagnostic and therapeutic. However, there is a growing number of recent reports showing the invaluable role of imaging on the diagnosis of Amyand hernias and associated complications. The correct and timely recognition of their imaging features including complications can optimize and expedite patient care by guiding diagnosis, treatment, and prognosis. Here, we report for the first time the radiological and pathological findings of a patient with a unique complicated Amyand hernia, which posed a diagnostic challenge for the clinical and radiological teams.

## Case report

An 84-year-old woman presented to the Emergency Department after a referral from her general practitioner with a lump in her right groin. She first noticed the lump, which had been increasing in size and tenderness, 2 weeks prior to her presentation. She was otherwise systemically well with no fever or any pain elsewhere. Her medical history included pernicious anaemia, hypertension, and pre-diabetes. She had a body mass index of 32 kg/m^2^. On examination an indurated and tender erythematous mass was palpated in the right groin. The rest of her examination was normal, including a soft and non-tender abdomen on palpation. Her blood tests were significant for a C-reactive protein of 52 mg/L [<6 mg/L] and a platelet count of 573 × 10^9^/L [160-370 × 10^9^/L]. Her white blood cell count was within the normal range at 10.3 × 10^9^/L [3.6-10.5 × 10^9^/L]. She was assessed by the general surgical team who gave a working diagnosis of cellulitis with an underlying abscess. An ultrasound examination of the groin was requested to look for an abscess, which the patient returned to the hospital to undergo the next day.

**Figure 1.**
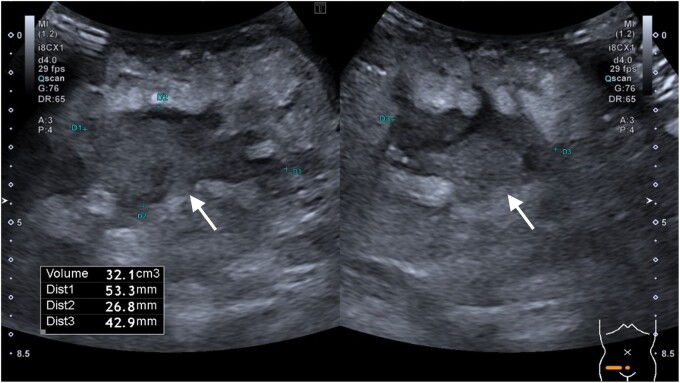
Transverse and longitudinal ultrasound images of the right groin showing an ill-defined fluid collection with surrounding echogenic fat (arrows).

The ultrasound examination showed an approximately 5 × 3 × 4 cm hypoechoic region in the subcutaneous fat overlying the right groin, which tracked to the labia majora (see Figure 1) This area was ill-defined with a surrounding rim of echogenic fat and mild increase in the Doppler signal. These findings were considered to represent an abscess and the patient was started on intravenous (IV) co-amoxiclav as well as fluids and analgesia.

**Figure 2.**
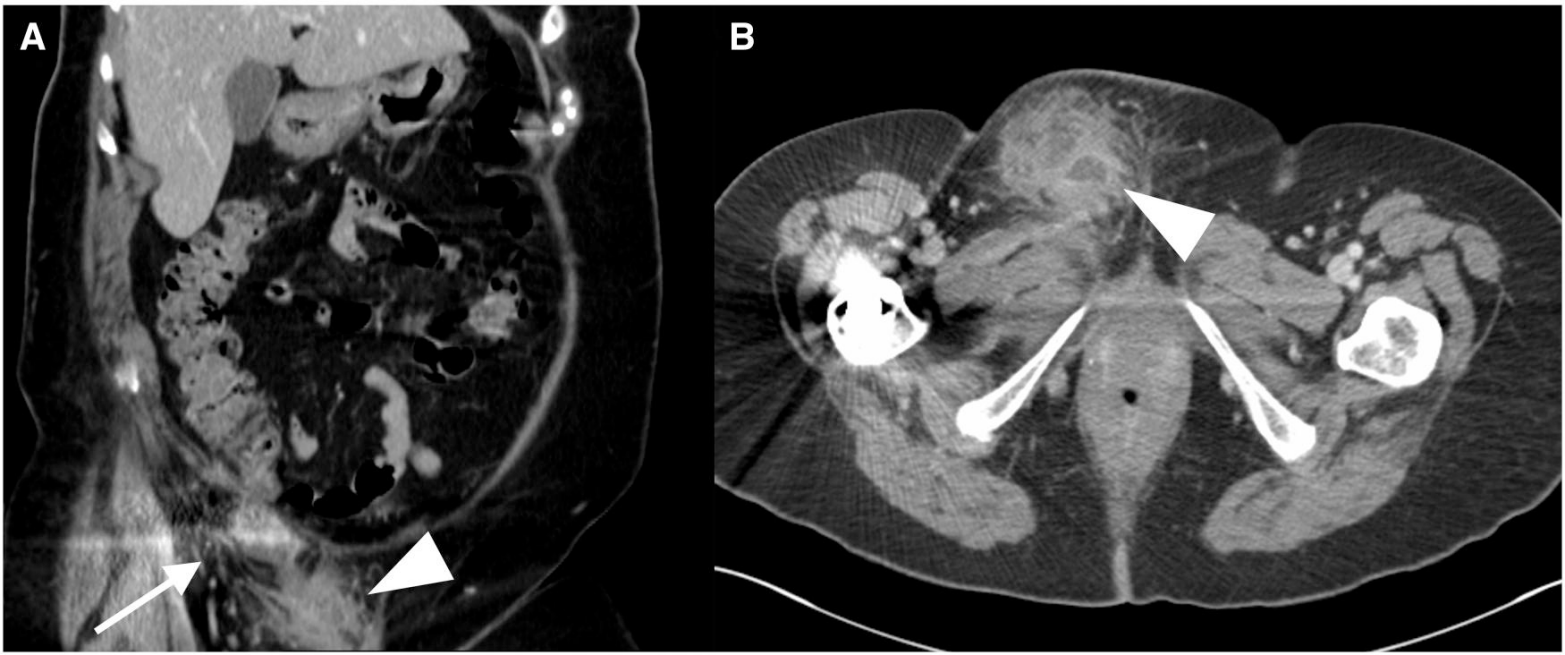
Contrast enhanced CT in the portal phase in coronal (A) and axial (B) planes demonstrating the right inguinal hernia containing the perforated dilated appendix (arrow) with an adjacent abscess tracking to the right labia majora (arrowhead).

**Figure 3.**
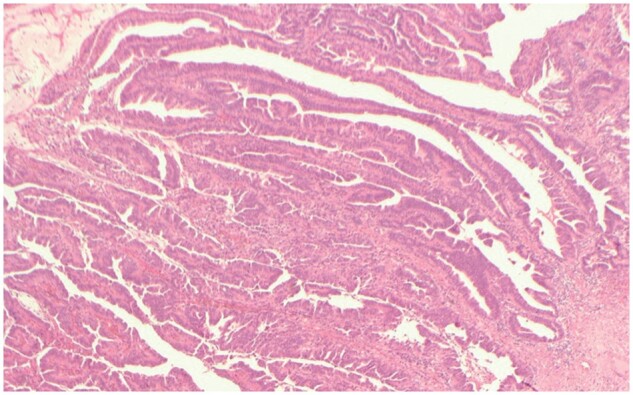
Microphotograph demonstrating the appendiceal moderately differentiated adenocarcinoma.

After 2 days of IV antibiotics, the patient was clinically stable and a CT scan of the abdomen and pelvis was performed for further assessment of the right groin. This study showed a dilated fluid-containing appendix passing into the right inguinal canal. At the tip of the inflamed appendix a collection with an ill-defined enhancing border was seen in the labia majora (see Figure 2), corresponding to the abscess seen on ultrasound. These findings were consistent with a perforated right Amyand hernia complicated with an abscess tracking superficially to the right labia majora. No further abnormalities were identified in the rest of the abdomen and pelvis, with no obstruction, free fluid or pneumoperitoneum. The patient underwent an open appendicectomy and right inguinal hernia repair. Pathological evaluation of the resected appendix and caecal pole was difficult due to the amount of firm fibrofatty tissue present in the sample but a presumed appendix with a normal (5 mm) calibre and evidence of perforation at its tip was identified. Histological analysis demonstrated a moderately differentiated invasive adenocarcinoma of the appendix extending to the serosal surface (see Figure 3). No malignancy was present in the resected lymph nodes. A CT of the thorax, abdomen, and pelvis with contrast was later performed with no evidence of metastatic disease.

## Discussion

An Amyand hernia can occur at any age (3 weeks-92 years old),^1^ however, the diagnosis is more frequent in children due to the patency of the processus vaginalis.^2^ Males are more commonly affected, with only a few cases reported in postmenopausal women,^1^ as in our case report. The majority of Amyand hernias are right indirect inguino-scrotal hernias, with the hernia sac passing through the deep inguinal ring lateral to the inferior epigastric vessels (this is in contrast to a direct inguinal hernia, which involves the hernia sac protruding through a defect in the posterior wall of the inguinal canal medial to the inferior epigastric vessels).^3^^,^^4^ When on the left side, this is often due to gut malrotation, situs inversus totalis, or a very mobile caecum.^4^

While the incidence of this type of hernia is rare, the appendix may remain within the inguinal hernia without symptoms or become inflamed, infected, strangulated or perforated with symptoms.^5–8^ Although incarceration of the appendix within an inguinal hernia does not necessarily lead to appendicitis, this combination of events is not uncommon.^9^ Several complications of Amyand hernias have been reported in the literature spanning from non-complicated appendicitis,^10^ to abscess formation,^11^ perforation and consequential necrotising fasciitis.^12^^,^^13^ As in our case report, only a few other cases of appendiceal cancer have been reported.^14–17^ However, our case is the first ever report of an appendiceal adenocarcinoma with superadded perforated appendicitis with abscess formation.

Not infrequently (in about 40% of the cases), patients with appendiceal tumours can present with acute appendicitis. However, appendiceal tumours are a rare entity, representing less than 0.5% of the gastrointestinal tract neoplasms.^18^ The most common type of appendiceal neoplasms is the carcinoid, making up 85% of appendiceal tumours.^17^ Appendiceal adenocarcinomas are an even rarer entity with an unclear incidence. The few existing studies in the field, report appendiceal neoplasms to occur in 1.4% of all post-appendicitis resected appendices, with 0.3% representing adenocarcinomas.^19^ When present, approximately 22% of the patients with appendiceal adenocarcinomas are believed to have metastatic disease at the time of diagnosis.^20^

The preoperative diagnosis of an Amyand hernia is often difficult and non-specific, with common complaints including sudden-onset periumbilical pain with localized tenderness in the right lower quadrant, combined with a tender irreducible mass in the inguinal or inguino-scrotal region.^10^^,^^21^ This presentation, however, often leads to the clinical impression of a strangulated hernia,^11^^,^^21^ which is typically managed surgically with no requirement for prior imaging. Further differential diagnoses include a strangulated omentocoele, inguinal adenitis and a Richter’s hernia, with an acute hydrocoele, epididymitis, and a testicular tumour with haemorrhage being additional considerations in male patients.^22^

Laboratory tests are not always helpful as they can be unremarkable. However, such as in our case, altered blood tests can point to the presence of complications like inflammation and/or abscess formation, which often require imaging to assist in the differential diagnosis.

As a rare entity, patients are frequently referred to imaging for the exclusion of collections or complications arising from strangulated hernias and not for exclusion of an Amyand hernia. Though, when performed, imaging can play an important role in diagnosing an Amyand hernia,^9^^,^^10^ as well as identify associated complications to aid the clinical management.

Ultrasound is an easy and inexpensive initial technique which can assess for the presence of collections and in experienced hands can diagnose an Amyand hernia,^23^ without the need for further cross-sectional imaging.^24^ On imaging, an Amyand hernia will appear as a blind-ending tubular structure passing into the inguinal canal, with or without inflammation. When inflamed, the appendix becomes enlarged, with various degrees of peri-appendicular fat stranding and inflammatory caecal thickening, depending on the extent of the local inflammation. Occasionally, an appendicolith might also be present. Common complications include perforation and collection formation. Appendiceal cancer in an Amyand hernia can be suspected on imaging depending upon the cancer size and the extent of inflammatory changes. In our case, the florid inflammatory changes within the right inguinal canal combined to the small size of the tumour made this diagnostic suspicion impossible. Presence of metastatic disease should raise concern for careful search for the primary, including in the appendix.

According to the Losanoff and Basson classification, Amyand hernias can be divided into 5 types based upon their imaging features. This classification is important as it provides guidance on surgical management.^25^ Type I refers to a normal appendix suggesting surgical reduction or appendicectomy with mesh hernioplasty.^26^ Type II is an acute appendicitis localized in the hernial sac: perform appendicectomy through hernia, with mesh hernia repair; associated with higher risk of mesh infection. Type III involves an acute appendicitis complicated by peritonitis: perform appendicectomy through laparotomy; hernioplasty decision should be made based upon the spread of sepsis. Type IV is an acute appendicitis with other abnormal pathology, implying that hernioplasty may be contraindicated if damage is too extensive. Type V occurs when the appendix is contained within a post-operative incisional hernia.

Mortality of an Amyand hernia has been reported to range from 14% to 30% and in most cases is linked to peritoneal spread of sepsis.^8^

## Learning points

- Diagnosis of an Amyand hernia remains challenging before radiological imaging is acquired.
- The use of ultrasound should always be considered as a first approach in the context of a painful inguinal mass.
- However, CT can provide invaluable information on the diagnosis, identification of complications, including co-existing cancer and metastatic disease or other conditions, and assist in surgical management.

## Data Availability

The datasets used and/or analysed during the current study are available from the corresponding author on reasonable request.
